# Patients with Schizophrenia Showed Worse Cognitive Performance than Bipolar and Major Depressive Disorder in a Sample with Comorbid Substance Use Disorders

**DOI:** 10.3390/jcm11226648

**Published:** 2022-11-09

**Authors:** Julia E. Marquez-Arrico, Alvaro Gonzalez-Sanchez, José Francisco Navarro, Rafael Penadés, Ana Adan

**Affiliations:** 1 Department of Clinical Psychology and Psychobiology, School of Psychology, University of Barcelona, Passeig de la Vall d’Hebrón 171, 08035 Barcelona, Spain; 2 Institute of Neurosciences, University of Barcelona, 08035 Barcelona, Spain; 3 Department of Psychobiology, School of Psychology, University of Málaga, Campus de Teatinos s/n, 29071 Málaga, Spain; 4 Barcelona Clínic Schizophrenia Unit (BCSU), Hospital Clínic Barcelona, 08036 Barcelona, Spain; 5 Institute of Biomedical Research August Pi i Sunyer (IDIBAPS), 08036 Barcelona, Spain; 6 Centro de Investigación Biomédica en Red Salud Mental (CIBERSAM), 28029 Madrid, Spain

**Keywords:** substance use disorders, schizophrenia, bipolar disorders, major depressive disorders, neurocognition, dual disorders

## Abstract

Comorbidity of substance use disorders (SUD) and severe mental illness (SMI) is highly frequent in patients, the most common diagnoses being schizophrenia (SZ), bipolar disorder (BD) and major depressive disorder (MDD). Since comorbidity has its own clinical features, and neurocognitive functioning is not always similar to psychiatric symptoms the present study explores the cognitive performance of patients with dual disorders. A neuropsychological battery of tests was used to assess 120 under treatment male patients, 40 for each group considered (SZ + SUD, BD + SUD and MDD + SUD) who were mainly polyconsumers. Significant differences (with premorbid IQ as a covariate) were found among the groups, with SZ + SUD having a worse performance in attention, verbal learning, short term memory and recognition. The consideration of a global Z score for performance evidenced an impaired neurocognitive pattern for SZ + SUD compared with BD + SUD and MDD + SUD. According to norms, all patients showed difficulties in verbal learning, short-term memory and recognition. Our research indicated that the neurocognitive functioning of dual disorder patients was influenced by the comorbid SMI, with SZ + SUD presenting major difficulties. Future studies should thoroughly explore the role of such difficulties as indicators or endophenotypes for dual schizophrenia disorders, and their usefulness for prevention and treatment.

## 1. Introduction

The coexistence of a psychiatric disorder and a substance use disorder (SUD) is highly prevalent [1,2] and comorbidity rates between the two diagnostic entities are estimated to be between 45–55% [3]. This phenomenon is called dual disorder and entails a more problematic sociodemographic and clinical profile compared to patients who have only a diagnosis of severe mental disorder or SUD [1,4]. Different studies have reported that the most severe and prevalent comorbid diagnoses of SUD in clinical samples are schizophrenia (SZ), bipolar disorder (BD) and major depressive disorder (MDD) [5,6].

The presence of an SUD has been associated in different studies with cognitive deficits in memory, attention, emotional processing, executive functioning and decision-making [7,8]. The study of cognition takes on special relevance in people with dual disorders since their influence on both treatment outcomes for SUD [9,10] and mental disorder has been evidenced [11,12]. In addition, for patients under treatment, knowledge of their own deficits or areas for improvement is a factor that is largely mediating therapeutic success [13].

Few studies have analysed the influence of dual diagnosis on neurocognition and, although most of the data point to worse performance of dual patients compared to those with only one diagnosis, there are also inconsistent findings. Some studies observe more severe deficits in patients with dual disorders compared to those with only severe mental illness (SMI) [12,14,15], while in other studies no differences were found between them [16,17]. Likewise, other works indicate that patients with dual disorders exhibit better cognitive performance [18,19] with respect to patients with a single diagnosis of SMI. In any case, the existence of possible implications of neurocognitive performance on the status, prognosis and outcomes of dual patients is certainly relevant [20].

The most investigated comorbidity has been the combination of SZ + SUD. Specifically, different studies report more impaired performance in patients with SZ + SUD, compared to SZ patients who are not substance users [21,22,23]. The functions observed with scores below normative values are premorbid IQ, current IQ, verbal learning, verbal working memory, processing speed and attention [23,24,25].

In the case of BD, few publications on patients with BD + SUD consistently point to worse performance in these patients compared to patients with BD without SUD in visual and verbal memory, conceptual reasoning, problem-solving skills and executive functioning [15,26,27]. Normalization of cognitive functioning in BD patients is possible, although the coexistence of SUD implies a less favourable recovery with deficits that may persist even when SUD treatment has been completed and the patient is euthymic [28,29].

Existing data on MDD + SUD patients point to the presence of lower scores on tests of verbal memory, immediate memory, processing speed and cognitive flexibility, compared to patients with only a diagnosis of MDD and to healthy controls [30,31]. However, in one study, no differences in cognitive performance were observed between MDD-only and SUD-only patients, who also had a normative performance in priming, short-term memory and verbal fluency [32].

Therefore, this work aims to explore, for the first time, the neurocognitive performance profile of a sample of patients with SUD and the three most prevalent comorbid SMI (SZ, BD and MDD). Our main hypothesis is that patients with dual disorders show similar cognitive functioning regardless of the type of comorbid severe mental disorder.

## 2. Materials and Methods

### 2.1. Participants and Procedure

A total sample of 120 male patients with SMI and comorbid SUD were included and considered in three groups, 40 for each psychiatric diagnosis: SZ + SUD, BD + SUD and MDD + SUD. All the participants were under psychiatric and/or psychological treatment, in outpatient or inpatient programs, at different public and private centres in the province of Barcelona (Catalonia) and were referred to our study from their treatment centres.

The inclusion criteria for our study were: (1) male sex (due to the higher prevalence rates of dual disorders for this sex in treatment centres as well as to control the possible SUD/SMI differences related to sex); (2) aged from 18 to 55 years old; (3) SUD diagnosis in initial remission phase (abstinence at least during a 3-month period, up to 12 months and confirmed by urinalysis), according to the Diagnostic and Statistical Manual of Mental Disorders criteria (DSM-5) [33]; (4) having the diagnosis of schizophrenia for the SZ + SUD group, bipolar disorder for the BD + SUD group and major depressive disorder for the MDD + SUD group, according to DSM-5 criteria for all cases; (5) being under treatment and stabilized. Participants were excluded from the study when meeting one of the following criteria: (1) presenting a disorder induced by substance use or medical illness according to DSM-5; (2) meeting DSM-5 criteria for other mental disorders; (3) presenting any physical and/or mental condition that could affect completing tasks. A psychologist from our research team individually administered the assessment protocol and participants were not economically compensated for their participation.

### 2.2. Instruments

#### 2.2.1. Sociodemographic and Clinical Measures

We designed an ad hoc structured interview to collect sociodemographic and clinical data such as age, marital status, educational level, living arrangements, SUD and psychiatric diagnosis age of onset, SUD relapses and psychopharmacological treatment, among others. In addition, the Structured Clinical Interview (SCID-I) for the DSM-IV-TR [34] was used to confirm SUD, SZ, BD and MDD diagnoses and to complete data collection. We applied the DSM-IV-TR version of the SCID-I because, at the time of assessment, the Spanish version of the DSM-5 was not yet available. Moreover, the Spanish version of the Drug Abuse Screening Test (DAST-20) was used to obtain an indicator of SUD severity following these cut-off points: 0 no addiction, 1–5 mild, 6–10 intermediate, 11–15 high and 16–20 severe [35].

The assessment of psychiatric symptoms according to the diagnosis was carried out through the Spanish version of the Positive and Negative Syndrome Scale (PANSS) [36] for the SZ + SUD group. The Spanish version of the Young Mania Rating Scale (YMRS) [37] was applied in the BD + SUD group to asses maniac symptoms and interpreted as follows: total score ≤ 12 indicates remission; 13–19 minimal symptoms; 20–25 mild mania; 26–37 moderate mania; and 38–60 severe mania. Finally, the Hamilton Depression Rating Scale (HDRS) in its Spanish version with 17 items [38] was used to assess depressive symptoms in the MDD + SUD group with scores from 0 to 7 indicating clinical remission; 8 to 13 mild depression; 4 to 18 moderate depression; 19–22 severe depression; and >23 severe depressive symptoms.

#### 2.2.2. Neurocognitive Performance Assessment Battery

Two subscales of the Wechsler Adult Intelligence Scale-Revised WAIS III [39] were used, the Vocabulary subtest was administered to assess the premorbid verbal IQ while the Digit Span Subtest (direct and indirect) was to assess the attention span [40]. For the correction and interpretation of these two tests, we applied the Spanish normative data available at the time of the assessment [39]. Verbal learning, memory processes, recall and recognition were explored with the Rey Auditory Verbal Learning Test (RAVLT) [41] and interpreted according to normative data [42]. Additionally, two neuropsychological measures were established with the RAVLT. The comparison between trails A5 and A7 was used to see how the participants recover information and the comparison between A7 and RecogA/15 was calculated to establish how the information is codified. Moreover, the Trail Making Test parts A and B (TMT-A and TMT-B) [43] were used and interpreted according to Spanish norms [44]. The TMT-A was administered as a measure of attention and processing speed, while the TMT-B provided a measure of cognitive flexibility and set-shifting.

Two computerized tests were also applied. On the one hand, the Wisconsin Card Sorting Tests (WSCT) [45] was applied as a measure of the ability to shift attention, problem solving, response maintenance, cognitive flexibility and frontal functioning. This test was interpreted according to norms taking into account age and years of education [45]. On the other hand, the Tower of Hanoi [46] in its four disks computerized version served as a measure of planning and problem-solving skills, and working memory was used. For the Tower of Hanoi task, there were no Spanish norms available at the time of the assessment, so their direct results were compared among the groups. All participants completed the full assessment battery.

### 2.3. Statistical Analysis

Descriptive statistics were calculated for the demographic and clinical variables for the three dual disorder groups, using ANOVA analyses for continuous data and chi-square test for categorical data. Means, standard deviation and percentages were calculated depending on the type of variable. All the results from the neurocognitive tasks (vocabulary, digits, RAVLT, TMT-A and B and WSCT) were transformed from direct to Z scores according to norms (considering sex, age and educational level), with the exception of the Tower of Hanoi since it has not any norms available for our population. The different cognitive functions considered were attention (Digits form WAIS III and A1 from RAVLT), verbal learning (total words from RAVLT), short-term memory (A6 from RAVLT), recognition (A/15 from RAVLT), processing speed (TMT-A) and cognitive flexibility (Wisconsin Sort Card Test and TMT-B).

The differences in neurocognitive performance among the groups were analysed through ANCOVA or MANCOVA depending on the task; repeated measures MANCOVA analysis were also used for RAVLT. For assessing global performance, following previous works [47,48,49] the Z scores for the different cognitive functions were all integrated into a global Z score as a measure of general performance. To evaluate the possible influence of sociodemographic variables (e.g., educational level, employment) as well as because it has also shown to be a variable with influence in cognitive performance [9,25], premorbid IQ (Vocabulary from WAIS III) was introduced as a covariable in all the analyses. We also estimated statistic partial eta squared (*η_p_*^2^) to measure the effect size, where a value of 0.01 was low, 0.04 moderate and 0.1 high [50]. All post hoc comparisons were Bonferroni corrected and data were analysed using the Statistical Package for the Social Sciences (SPSS version 25.0, SPSS Inc., Chicago, IL, USA). All the tests were considered bilaterally with a type I error established at 5%.

## 3. Results

### 3.1. Sociodemographic Data and Clinical Variables Related to the Comorbid Severe Mental Illness

Analysis of the sociodemographic and clinical data (see Table 1) indicated that the mean age of the total sample of patients included was 37.76 years (*SD* = 7.61), with the age of the SZ + SUD group being lower than that of the MDD + SUD group (*p* = 0.012). Significant differences between groups were found in the variables of family situation, living arrangements and economic situation. In this regard, the SZ + SUD group contributed the highest rates of patients without children (*p* = 0.001), and of sharing a place of residence (*p* = 0.001), while the MDD + SUD group showed the highest number of patients in a situation of unemployment (*p* = 0.001), although with fewer disability pensions (*p* = 0.001).

Regarding clinical characteristics (see Table 2), the analysis of variables linked to SMI indicated that the mean age at onset was 27.22 years (*SD* = 8.72), with the MDD + SUD group showing the latest age (*p* < 0.001). In relation to medication, the SZ + SUD group showed the highest amount of daily medication compared to the MDD + SUD group (*p* = 0.021), with the prescription of typical (*p* = 0.019) and atypical antipsychotics (*p* < 0.001) specific for their SMI. Likewise, the BD + SUD group presented the highest percentage of mood stabilizers prescription (*p* < 0.001) while the MDD + SUD group stood out for the higher use of antidepressants (*p* = 0.017). In the case of psychiatric symptoms, the direct PANSS scale scores in the SZ + SUD group were in the following percentiles: 5 for positive symptoms, 15 for negative symptoms, 45 for the composite scale and 10 for general symptomatology. The BD + SUD group showed values on the YMRS and HDRS scales that placed them without significant manic or active depressive symptomatology, while the MDD + SUD group had a higher HDRS score than BD + SUD (*p* < 0.001), which placed them in the mild depressive symptoms range.

In reference to the clinical variables related to SUD (see Table 3), the age of onset of the SUD was 18.79 years (*SD* = 6.67) with no differences between the groups. Polydrug use was the main pattern of substance use in the total sample (56.7%), with the SZ + SUD group having the highest rate of polydrug patients of the three groups (*p* = 0.015). The main psychoactive substances consumed were cocaine, alcohol and cannabis, with some significant differences among the groups. The BD + SUD group had the lowest percentage of cocaine use (*p* = 0.001) while the SZ + SUD group had the highest percentages of cannabis use (*p* = 0.004), hallucinogens (*p* = 0.024) and anxiolytic/hypnotic-sedative drugs (*p* = 0.017). The SZ + SUD and BD + SUD groups had the highest daily cigarette consumption and a higher score on the Fagerström dependence test than the MDD + SUD group (*p* ≤ 0.029). Significant differences were observed among the groups in severity of addiction (DAST-20); the means of the SZ + SUD and MDD + SUD groups were within the high severity category differing from the BD + SUD group (*p* = 0.009) with mild severity. The abstinence period of the total sample was 9.69 months on average (*SD* = 7.31) and the groups did not differ from each other.

### 3.2. Comparisons of Neurocognitive Performance among the Groups and Considering Normative Data

The estimation of the verbal premorbid IQ (vocabulary test) did not provide differences among groups (*F*_(2,119)_ = 1.687; *p* = 0.189; *η_p_*^2^ = 0.028), with Z values in the three cases close to the normative mean: SZ + SUD = −0.27 ± 0. 13; SUD + BD = 0.26 ± 0.15, and SUD + MDD = 0.29 ± 0.12. The result in the digit test was similar among the different groups (*F*_(2,119)_ = 1.647; *p* = 0.197; *η_p_*^2^ = 0.028) with group Z-scores and standard error of: SZ + SUD = −0.41 ± 0.14, BD + SUD = −0.28 ± 0.15, and MDD + SUD= −0.05 ± 0.13. As can be seen in Table 4, significant differences were obtained among groups in the performance of the RAVLT and TMT-B tasks. In the RAVLT, SZ + SUD presented the lowest performance in total words compared with BD + SUD and MDD + SUD (*p* < 0.01 in both cases) while these last two groups did not show any significant differences between them (*p* = 1.000). Recognition was worse for SZ + SUD compared to MDD + SUD (*p* = 0.002) while the BD + SUD group was in an intermediate position with no differences compared with SZ + SUD and BD + SUD (*p* ≥ 0.103).

**Figure 1:**
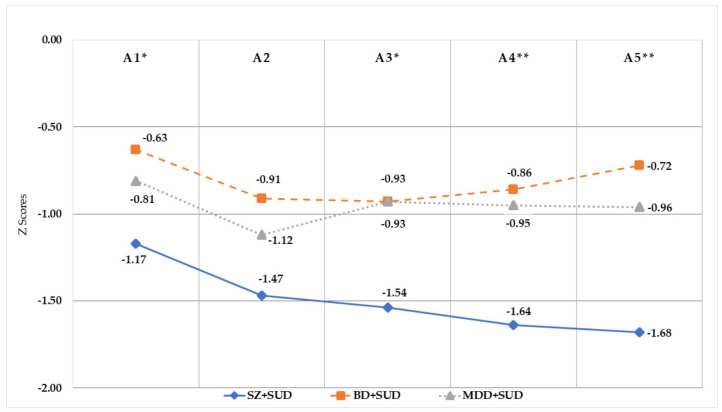
Learning curve in Z scores for the Rey Auditory Verbal Learning Test trials by groups. SZ + SUD: schizophrenia with comorbid substance use disorder; BD + SUD: bipolar disorder with comorbid substance use disorder; MDD + SUD: major depressive disorder and comorbid substance use disorder. * *p* < 0.05; ** *p* < 0.01.

On the other hand, the repeated measures analysis of the RAVLT provided significant differences among the groups in the learning curve (see Figure 1). The SZ + SUD group obtained the lowest performance (*F*_(2,119)_ = 4.078; *p* = 0.004; *η_p_*^2^ = 0.126), with respect to both BD + SUD (*p* = 0.003) and MDD + SUD (*p* = 0.006). The learning curve (trials A1 to A5) of the BD + SUD and MDD + SUD groups was similar (*p* = 1.000). Trial A1 of the RAVLT presented differences (*F*_(2,119)_ = 3.418; *p* = 0.036; *η_p_*^2^ = 0.056), with the performance of the SZ + SUD being lower than BD + SUD (*p* = 0.034), while the MDD + SUD group was in an intermediate position. No differences were observed among the groups in trial A2 (*F*_(2,119)_ = 2.844; *p* = 0.062; *η_p_*^2^ = 0.040). On the other hand, trials A3, A4 and A5 showed a worse execution of the task for the SZ + SUD group (*F*_(2,119)_ ≥ 4.271; *p* ≤ 0.016; *η_p_*^2^ ≥ 0.070), their performance was inferior to that of the BD + SUD (*p* ≤ 0.037) and MDD + SUD (*p* ≤ 0.022) groups. Considering the normative data, all three groups performed below average on the learning curve (see Figure 1). Moreover, no differences among the groups were observed in the comparison of A5 vs. A7 trials for the delayed recall (*F*_(2,119)_ = 1.80; *p* = 0.170; *η_p_*^2^ = 0.030) nor for trial A7 vs. Rec A/15 for encoding and recall with cues (*F*_(2,119)_ = 0.916; *p* = 0.403; *η_p_*^2^ = 0.016).

Moreover, TMT-A results were not associated with significant differences among groups, although in all three cases processing speed was slower than that of the normal population. In the case of TMT-B, it was the BD + SUD group that presented worse cognitive flexibility with respect to MDD + SUD (*p* = 0.011), with the SZ + SUD group in an intermediate position and with no differences with respect to BD + SUD or MDD + SUD (*p* ≥ 0.275).

Performance on the WSCT and Tower of Hanoi tasks (see Table 4) did not provide significant differences among groups. In the case of the WSCT, the performance of the three groups was also within normative values (Z between 1 and −1), with the exception of the number and percentage of perseverative errors where the performance of all three groups was above normative values (Z ≥ 1.5).

**Figure 2:**
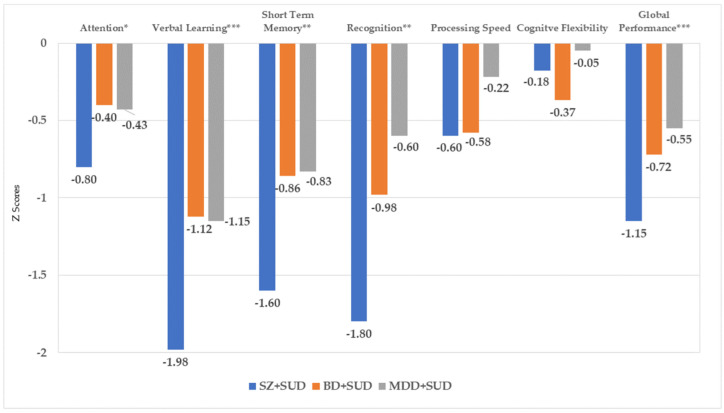
Z scores on the assessed cognitive domains for each of the groups. *Note:* Attention is composed by digits (WAIS III) and A1 (RAVLT). Verbal learning refers to total words (RAVLT). Short term memory is composed by A6 (RAVLT). Recognition refers to Recog. list A/15 (RAVLT). Processing speed is TMT-A. Cognitive flexibility was calculated form Wisconsin Sort Card Test and TMT-B. SZ + SUD: schizophrenia with comorbid substance use disorder; BD + SUD: bipolar disorder with comorbid substance use disorder; MDD + SUD: Major depressive disorder and comorbid substance use disorder. * *p* < 0.05; ** *p* < 0.01; *** *p* ≤ 0.001.

The results in Z scores according to the cognitive functions assessed (see Figure 2) indicated differences among groups for attention (*F*_(2,119)_ = 3.933; *p* = 0.037; *η_p_*^2^ = 0.051), verbal learning (*F*_(2,119)_ = 7.087; *p* = 0.001; *η_p_*^2^ = 0.115), short-term memory (*F*_(2,119)_ = 6.653; *p* = 0.002; *η_p_*^2^ = 0.109) and recognition (*F*_(2,119)_ = 6.252; *p* = 0.003; *η_p_*^2^ = 0.103), as well as in global performance (*F*_(2,119)_ = 7.332; *p* = 0.001; *η_p_*^2^ = 0.19). In all cases, the SZ + SUD group presented the worst performance and altered values (Z ≤ −1.5) in verbal learning, short-term memory and recognition. In contrast, no differences were found in the domains of processing speed (*F*_(2,119)_ = 1.198; *p* = 0.306; *η_p_*^2^ = 0.022) and cognitive flexibility (*F*_(2,119)_ = 2.100; *p* = 0.127 *η_p_*^2^ = 0.037).

## 4. Discussion

Contrary to our expectations, dual disorders showed different cognitive profiles in patients with comorbid SZ, BD and MDD. Thus, the neurocognitive functioning of dual disorders was influenced by the comorbid SMI, with the SZ + SUD group showing higher impairments. To the best of our knowledge, this study provides the first data on the possible differences in cognitive performance for patients with dual disorders according to their diagnosis of SMI. The sociodemographic and clinical characteristics of the patients in our sample are in line with previous studies [51,52] with the SZ + SUD group showing a worse social and clinical situation. Such characteristics are relevant since in previous studies they have been related to poorer adherence to the treatment [53], clinical course and prognosis [54]. The results for the type of drug used in the total sample and for each group reflect the current state of SUDs in our population [55,56] with a high prevalence of alcohol, cocaine and cannabis use, as well as the type of patients receiving SUD treatment [55,57].

The neurocognitive performance findings are consistent with previous studies [15,16,26,30] since a performance below normative means was observed in our sample regardless of the comorbidity combination. It should be specified, however, that patients with SZ + SUD are those who presented the worst performance in functions such as attention, learning ability and memory. Our results on the performance of each group extend previous findings [22,24,25] to patients in the early remission phase of SUD and to those with an SZ + SUD diagnosis with stabilized psychotic symptomatology.

Through the analysis of the learning curve, it has been shown that patients with SZ + SUD perform worse as the test progresses without the beneficial effect of repetition or verbal learning, and this performance pattern is not found in patients with BD + SUD or MDD + SUD (Figure 1). All this supports the evidence that verbal declarative memory is one of the most altered functions in patients with SZ + SUD and could be considered an indicator or endophenotype of schizophrenia spectrum disorders [23,58].

Regarding the results in executive functions measured by the TMT-B and WSCT, the performance of the groups was similar to previously published data [47,59] as well as to normative values. Performance on the TMT-B test was worse in BD + SUD patients, which has also been observed in BD patients without substance use [60]; in the future, this finding should be explored as a possible trait or cognitive correlate differentiating BD + SUD patients from those with MDD + SUD.

On the other hand, performance on the WSCT was similar among the different groups and with respect to normative data. This is not consistent with previous studies that found a worse performance on executive functions for patients with SUD [61] and/or SZ [17], BD [27] or MDD [30]. However, the study by Verdejo-García and Pérez-García (2007) which suggested that WSCT would not be an adequate test to discriminate performance between patients with SUD and healthy controls, should be considered. Our data are in this line and extend such observations to dual patients regardless of the comorbid SMI considered. A possible explanation for this normative executive performance in our patients may be that they are in the early remission phase of SUD, being well known for the benefit of the months of abstinence for the recovery of executive functions [62,63].

A noteworthy finding was the better performance of the groups in the number and percentage of perseverative WSCT errors compared to the general population. This better performance was not reflected in the number of categories completed, attempts to complete the first category or in the learning to learn score. Perseverative errors occur when the subject gives a response adjusted to the previously learned strategy [45]. Thus, the lower number with respect to the normal population would indicate a quick change in criterion or an unreflective way of responding to the test. This result could also be valued as an indicator of functional impulsivity, related to the rapid generation of ideas and decision-making [64].

At the global performance level, the score that includes all the evaluated domains shows differences among groups, being the patients with SZ + SUD those who presented the worst performance compared with BD + SUD and MDD + SUD patients. Although all three groups obtained scores below the normative mean, it is the score of the SZ + SUD group that can be considered altered in neuropsychological terms. Considering the presence of the two diagnoses in the patients studied, an important alteration in their cognitive performance could be expected, which does not occur in the BD + SUD and MDD + SUD groups. A possible explanation is found in the theoretical framework that points out a lower biological vulnerability in dual disorders with respect to SMI without comorbidity [19,47]. Patients with dual disorders would start from a better premorbid functioning that would have allowed them a level of social functioning to acquire illegal substances of abuse [65] and have ended up developing the SMI possibly as a result of drug abuse.

The present study has strengths and limitations. One of its strengths is that it represents a first approximation of the cognitive performance profile of patients with dual disorders according to their diagnosis of SMI, exploring their cognitive functions with a wide battery of tests. Likewise, having considered Z scores according to scales has allowed comparison with normative data, and controlling for the effect of age on performance. However, the study has limitations such as having a sample with a majority pattern of polydrug use that does not allow discriminating of the possible differential effect of the psychoactive substance and not controlling the possible effect of medication among groups. The differences among the groups in the type of medication are according to the expected (since it is related to the type of SMI) but it might influence their cognitive performance. Oncoming studies should consider the type and dose of pharmacological treatment and their possible influence on patients with SMI and SUD. Moreover, we did not include women to assess the different profiles according to sex or longitudinal measures that allow analysing of the evolution of cognitive functions as the treatment received by the patient progresses. In this sense, including both inpatients and outpatients in or sample might be considered a limitation of this work. Some evidence shows that inpatients have worse cognitive performance than outpatients [66] while other data indicate that such differences are not significant or observed [67]. Future studies should include larger sample sizes that allow analyses to explore the differences associated with the type of treatment beyond the SMI. Our data emphasize that the pattern of cognitive performance observed in SZ + SUD patients is more similar to the deficits found in SZ than in SUD, pointing to the need to assess neurocognition in these dual patients. This may help to incorporate rehabilitation strategies during treatment, which seems to be of particular relevance in the SZ + SUD group, as is the case with SZ diagnosis alone [68]. Likewise, the impairment of memory functions for SZ + SUD should be considered in the design of treatment programs as it implies a limitation in the ability to learn and recognize information that may negatively affect treatment adherence [69].

## 5. Conclusions

This study explored the neurocognitive profiles in the three most severe and prevalent psychiatric diagnoses in patients with dual disorders. The SZ + SUD group presented an altered performance in memory, short-term memory, verbal learning and global performance, supporting previous studies that point to alterations in declarative memory as putative indicators or endophenotypes of SZ spectrum disorders. The neurocognitive performance profile in patients with SZ + SUD points to a greater vulnerability, emphasizing the need for its evaluation and consideration for incorporating cognitive rehabilitation strategies that may be essential for the success of the therapeutic approach with these patients.

## Figures and Tables

**Table 1:** Sociodemographic data for the three groups of patients. Means, standard deviation, percentages and statistical contrasts (ANOVA and chi-square test).

Sociodemographic Data	SZ + SUD (*N* = 40)	BD + SUD (*N* = 40)	MDD + SUD (*N* = 40)	Contrasts
Age (years)	34.93 ± 7.71	38.55 ± 8.59	39.80 ± 5.55	*F*_(2,119)_ = 4.68 *
Marital status				χ^2^_(2)_ = 12.59
Single	82.5%	55.0%	50.0%	
Married/stable partner	15.0%	42.5%	45.0%	
Separated/divorced	2.5%	2.5%	5.0%	
Family situation				χ^2^_(1)_ = 9.45 **
Without children	82.5%	62.5%	50.0%	
With children	17.5%	37.5%	50.0%	
Living arrangements				χ^2^_(3)_ = 21.87 ***
Alone	7.5%	17.5%	5.0%	
Sharing	67.5%	62.5%	47.5%	
Therapeutic community	15.0%	20.0%	47.5%	
Supported accommodation	10.0%	0%	0%	
Economic situation				χ^2^_(3)_ = 35.37 ***
Working	12.5%	10.0%	12.5%	
Unemployed	20.0%	20.0%	57.5%	
Under sick leave	7.5%	5.0%	17.5%	
Disability pension	60.0%	65.0%	12.5%	
Years of schooling	9.88 ± 2.43	11.25 ± 3.26	10.45 ± 2.05	*F*_(2,119)_ = 2.76

BD + SUD: bipolar disorder with comorbid substance use disorder; MDD + SUD: major depressive disorder with comorbid substance use disorder; SZ + SUD: schizophrenia with comorbid substance use disorder. * *p* < 0.05; ** *p* < 0.01; *** *p* < 0.001.

**Table 2:** Clinical data for the three groups of patients regarding psychiatric diagnosis. Means, standard deviation, percentages and statistical contrasts (ANOVA and chi-square test).

Clinical Data	SZ + SUD (*N* = 40)	BD + SUD (*N* = 40)	MDD + SUD (*N* = 40)	Contrasts
SMI age of onset (years)	23.88 ± 7.31	25.95 ± 8.82	31.83 ± 8.12	*F_(_*_2,119)_ = 10.34 ***
History of suicide attempts	45.0%	40.0%	47.5%	χ^2^_(1)_ = 0.47
Pharmacological treatment ^a^				
Quantity of medications per day	3.36 ± 1.56	3.12 ± 1.77	2.33 ± 1.64	*F*_(2,119)_ = 4.13 *
Typical antipsychotic	28.2%	10.0%	2.6%	χ^2^_(1)_ = 15.19 *
Atypical antipsychotic	94.6%	65.0%	22.5%	χ^2^_(1)_ = 44.89 ***
Mood stabilizers	41.0%	67.5%	32.4%	χ^2^_(1)_ = 26.02 ***
Anxiolytics	43.6%	35.0%	39.8%	χ^2^_(1)_ = 2.14
Antidepressants	33.3%	46.2%	71.8%	χ^2^_(1)_ = 15.48 *
Anticholinergic	25.6%	2.5%	0%	χ^2^_(1)_ = 18.25 ***
Alcohol-aversive-agent	25.6%	22.5%	25.6%	χ^2^_(1)_ = 0.14
Other psychotropics	13.2%	12.5%	17.9%	χ^2^_(1)_ = 2.24
Chlorpromazine equivalent dose (mg)	422.30 ± 35.22	138.81 ± 3.27	43.15 ± 34.27	*F*_(2,119)_ = 31.79 ***
PANSS positive	12.34 ± 6.20			
PANSS negative	15.07 ± 7.54			
PANSS composite	−2.65 ± 5.83			
PANSS general psychopathology	30.71 ± 11.62			
HDRS total score		7.06 ± 5.17	11.10 ± 5.28	*F*_(1,79)_ = 12.39 ***
YMRS total score		3.16 ± 3.13		

BD + SUD: bipolar disorder with comorbid substance use disorder; HDRS: Hamilton depression rating scale; MDD + SUD: major depressive disorder with comorbid substance use disorder; PANSS: positive and negative syndrome scale; SMI: severe mental illness; SUD: substance use disorder; SZ + SUD: schizophrenia with comorbid substance use disorder; YMRS: Young mania rating scale. ^a^ Percentages will not equal 100 as each patient may have taken more than one substance/more than one drug. * *p* < 0.05; *** *p* < 0.001.

**Table 3:** Clinical data for the three groups of patients regarding substance use and substance use disorder. Means, standard deviation, percentages and statistical contrasts (ANOVA and chi-square test).

Clinical Data	SZ + SUD (*N* = 40)	BD + SUD (*N* = 40)	MDD + SUD (*N* = 40)	Contrasts
SUD age of onset (years)	17.50 ± 5.19	20.33 ± 7.51	18.55 ± 6.94	*F*_(2,119)_ = 3.39
SUD duration (years)	17.49 ± 7.57	18.23 ± 9.10	21.25 ± 8.59	*F*_(2,119)_ = 2.12
Quantity of substances used	3.74 ± 1.44	2.54 ± 1.19	2.95 ± 1.41	*F*_(2,119)_ = 7.55 ***
Polydrug use	82.5%	37.5%	50.0%	χ^2^_(1)_ = 38.67 *
Type of substance ^a^				
Cocaine	97.5%	65.0%	82.5%	χ^2^_(1)_ = 14.14 ***
Alcohol	75.0%	87.5%	90.0%	χ^2^_(1)_ = 3.46
Cannabis	82.5%	47.5%	57.5%	χ^2^_(1)_ = 11.09 **
Ecstasy	17.5%	10.0%	5.0%	χ^2^_(1)_ = 3.27
Hallucinogens	40.0%	15.0%	20.0%	χ^2^_(1)_ = 7.46 *
Opioids	30.0%	12.5%	25.0%	χ^2^_(1)_ = 3.72
Anxiolytic/hypnotic-sedative	32.5%	10.0%	12.5%	χ^2^_(1)_ = 8.12 *
Daily cigarettes per day	21.82 ± 11.29	19.35 ± 9.13	13.83 ± 7.36	*F*_(2,119)_ = 7.61 ***
Fagerström total score	6.10 ± 2.60	5.03 ± 2.86	4.26 ± 2.41	*F*_(2,119)_ = 4.85 **
DAST-20 total score	12.89 ± 3.03	11.17 ± 4.98	13.47 ± 4.04	F_(2,119)_ = 2.35
Severity of addiction				χ^2^_(3)_ = 20.47 **
Low	3.7%	20.8%	3.3%	
Mild	14.8%	33.4%	16.7%	
High	70.4%	25.0%	50.0%	
Severe	11.1%	20.8%	30.0%	
Abstinence period (months)	11.13 ± 5.68	9.70 ± 6.69	8.22 ± 5.72	*F*_(2,119)_ = 0.74
Quantity of relapses	1.60 ± 3.38	0.90 ± 1.85	0.75 ± 1.30	*F*_(2,119)_ = 1.53

BD + SUD: bipolar disorder with comorbid substance use disorder; DAST-20: drug abuse screening test; MDD + SUD: major depressive disorder with comorbid substance use disorder; SUD: substance use disorder; SZ + SUD: schizophrenia with comorbid substance use disorder. ^a^ Percentages will not equal 100 as each patient may have taken more than one substance/more than one drug. * *p* < 0.05; ** *p* < 0.01; *** *p* < 0.001.

**Table 4:** Results for the three groups of patients in: RAVLT (Rey Auditory Verbal Learning Test), Trial Making Test (TMT A and B), Wisconsin Card Sorting Tests (WCST) and Tower of Hanoi. Means (in Z scores), standard error and statistical contrasts of the MANCOVA (Multiple Analysis of Covariance).

	SZ + SUD (*N* = 40)	BD + SUD (*N* = 40)	MDD + SUD (*N* = 40)	MANCOVA	
				*F*_(2,119)_	*η_p_* ^2^
RAVLT					
Total words	−2.00 ± 0.18	−1.10 ± 0.19	−1.11 ± 0.17	7.60 ***	0.119
Recog. list A/15	−1.80 ± 0.24	−0.98 ± 0.25	−0.60 ± 0.23	6.25 **	0.097
Trial Making Test					
TMT-A	−0.60 ± 0.19	−0.58 ± 0.20	−0.22 ± 0.18	1.58	0.027
TMT-B	−0.70 ± 0.17	−1.02 ± 0.18	−0.27 ± 0.16	4.42 *	0.073
WSCT					
Categories completed	5.53 ± 0.21	5.40 ± 0.19	5.43 ± 0.20	0.108	0.002
Total errors	19.73 ± 2.27	20.72 ± 2.25	20.55 ± 2.26	0.054	0.001
Z scores	0.76 ± 0.19	0.50 ± 0.18	0.45 ± 0.17	0.786	0.013
Percentage of errors	19.25 ± 1.55	19.10 ± 1.53	19.90 ± 1.54	0.076	0.001
Z scores for percentage	0.73 ± 0.20	0.62 ± 0.18	0.51 ± 0.17	0.359	0.006
Perseverative errors	5.46 ± 0.98	6.22 ± 0.97	6.67 ± 0.96	0.386	0.007
Z scores	2.19 ± 0.22	1.69 ± 0.21	1.59 ± 0.20	2.197	0.036
Percentage perseverative errors	4.71 ± 0.75	5.16 ± 0.74	5.90 ± 0.76	0.645	0.011
Z scores for percentage	2.45 ± 0.19	2.10 ± 0.20	1.95 ± 0.21	1.584	0.027
Tower of Hanoi					
N° of movements	25.46 ± 1.71	25.10 ± 1.78	29.05 ± 1.72	1.589	0.028
Errors	1.25 ± 0.39	1.49 ± 0.40	1.36 ± 0.39	0.910	0.002
Response time	211.35 ± 22.26	202.55 ± 23.27	246.42 ± 22.38	1.055	0.019

BD + SUD: bipolar disorder with comorbid substance use disorder; MDD + SUD: major depressive disorder with comorbid substance use disorder; SZ + SUD: schizophrenia with comorbid substance use disorder; *η_p_*^2^: Partial eta squared. * = *p* < 0.05; ** = *p* < 0.01; *** = *p* < 0.001.

## Data Availability

The data presented in this study are available on request from the corresponding author.
